# Durability of three types of dual active ingredient long-lasting insecticidal net compared to a pyrethroid-only LLIN in Tanzania: methodology for a prospective cohort study nested in a cluster randomized controlled trial

**DOI:** 10.1186/s12936-022-04119-4

**Published:** 2022-03-19

**Authors:** Jackline L. Martin, Louisa A. Messenger, Franklin W. Mosha, Eliud Lukole, Jacklin F. Mosha, Manisha Kulkarni, Thomas S. Churcher, Ellie Sherrard-Smith, Alphaxard Manjurano, Natacha Protopopoff, Mark Rowland

**Affiliations:** 1 Kilimanjaro Christian Medical University College, Moshi, United Republic of Tanzania; 2 National Institute for Medical Research-Mwanza Center, Mwanza, United Republic of Tanzania; 3 London School of Hygiene and Tropical, London, UK; 4 University of Ottawa, Ottawa, Canada; 5 Imperial College, London, UK

**Keywords:** Bio-efficacy, Textile durability, Malaria vectors, Insecticide resistance, Pyriproxyfen, Chlorfenapyr, Piperonyl butoxide, Royal Guard, Interceptor G2, Olyset plus, Interceptor, Tanzania, Experimental hut trial, Long-lasting insecticidal net

## Abstract

**Background:**

Progress achieved by long-lasting insecticidal nets (LLINs) against malaria is threatened by widespread selection of pyrethroid resistance among vector populations. LLINs with non-pyrethroid insecticides are urgently needed. This study aims to assess the insecticide and textile durability of three classes of dual-active ingredient (A.I.) LLINs using techniques derived from established WHO LLIN testing methods to set new standards of evaluation.

**Methods:**

A WHO Phase 3 active ingredients and textile durability study will be carried out within a cluster randomized controlled trial in 40 clusters in Misungwi district, Tanzania. The following treatments will be evaluated: (1) Interceptor^®^G2 combining chlorfenapyr and the pyrethroid alpha-cypermethrin, (2) Royal Guard^®^ treated with pyriproxyfen and alpha-cypermethrin, (3) Olyset™ Plus which incorporates a synergist piperonyl butoxide and the pyrethroid permethrin, and (4) a reference standard alpha-cypermethrin only LLIN (Interceptor^®^). 750 nets will be followed in 5 clusters per intervention arm at 6, 12, 24 and 36 months post distribution for survivorship and hole index assessment. A second cohort of 1950 nets per net type will be identified in 10 clusters, of which 30 LLINs will be withdrawn for bio-efficacy and chemical analysis every 6 months up to 36 months and another 30 collected for experimental hut trials every year. Bio-efficacy will be assessed using cone bioassays and tunnel tests against susceptible and resistant laboratory strains of *Anopheles gambiae *sensu stricto. Efficacy of field-collected nets will be compared in six experimental huts. The main outcomes will be *Anopheles* mortality up to 72 h post exposure, blood feeding and egg maturation using ovary dissection to assess impact on fecundity.

**Conclusions:**

Study findings will help develop bio-efficacy and physical durability criteria for partner A.I., in relation to the cRCT epidemiological and entomological outcomes, and refine preferred product characteristics of each class of LLIN. If suitable, the bioassay and hut outcomes will be fitted to transmission models to estimate correlation with cRCT outcomes.

*Trial registration number*: NCT03554616.

**Supplementary Information:**

The online version contains supplementary material available at 10.1186/s12936-022-04119-4.

## Background

Long-lasting insecticidal nets (LLINs) are the primary method of malaria control in sub-Saharan Africa. The World Health Organization (WHO) estimates that over 50% of the population now sleeps under LLINs. This has helped to reduce malaria incidence by 42% and mortality by 66% in Africa over the last 15 years [1]. Until recently, pyrethroids were the only type of insecticide used routinely on LLINs. The rapid spread of pyrethroid resistance in vector populations threatens to reverse the success achieved so far [2]. Several studies have demonstrated that LLINs are becoming less effective at killing mosquitoes in areas of high resistance compared to before [3, 4].

The first new type of LLIN developed to control resistant mosquitoes is a combination LLIN containing permethrin and the synergist piperonyl butoxide (PBO), which inhibits cytochrome P450 oxidases responsible for metabolic resistance [5]. A community-based cluster randomized controlled trial (cRCT) conducted in north-western Tanzania (Kagera region) demonstrated a reduction in the prevalence of malaria by 44% in the pyrethroid-PBO LLIN arm (Olyset™ Plus) compared to the standard pyrethroid LLIN after one year and by 33% after two years [6, 7]. Based on this study, the WHO recognized the improved public health value of the pyrethroid-PBO LLIN in areas of high resistance and provided interim recommendation for pyrethroid PBO LLINs [8]. Since then, two dual-active ingredient (dual-A.I.) LLINs (Royal Guard^®^ and Interceptor^®^ G2) have been evaluated in WHO Phase I and II trials [9] and have shown promise compared to standard LLINs against pyrethroid resistant vectors. Each of these putative first-in-class LLINs are required by the WHO to undergo cRCTs versus standard LLINs to demonstrate, unequivocally, evidence of improved malaria control effect [10]. Two such community cRCTs are currently underway in Misungwi, Tanzania [11] and in Cove, Benin [12].

For each first-in-class LLIN, in addition to cRCTs, assessment of the quality, long lasting entomological efficacy and safety is also required for each putative vector control product submitted to the WHO [10]. Whether the LLIN product is a novel first-in-class LLIN, or a generic second-in-class LLIN, the LLIN should be evaluated in three different phases [9]. After LLIN assessment for insecticidal activity and wash-durability in the laboratory (Phase I), the bio-efficacy and safety of these nets are evaluated against host-seeking mosquitoes in the presence of human occupants, under realistic household conditions, in experimental hut trials (Phase II) [13, 14]. Thereafter, bio-efficacy, attrition and physical durability of nets are monitored in the community over 3 years in large-scale field trials requiring nets to be sampled from households and evaluating them for net integrity (hole index) and attrition (Phase III) [9]. Collectively, the data from the three phases are then reviewed by WHO for pre-qualification decision [10].

While community cRCTs provide definitive epidemiological evidence for the establishment of new product classes of LLIN, questions remain whether cRCTs should be the primary mechanism to generate malaria vector control evidence [15]. It has been proposed that entomological evidence generated by experimental hut trials, if used to parameterize malaria transmission models, may be adequate to make to this judgement [2], as the cost of EHTs are much lower than cRCTs and much shorter in duration than the 2 years needed for cRCT which may delay the introduction of new tools and disincentivize investment in new active ingredients [16].

In some respects, experimental huts are ideal for measuring the natural behaviour and killing of mosquitoes in the home as there is no manipulation of the host-seeking mosquito and no interference with the resting time of the blood-fed stage. Some malaria transmission models make use of LLIN Phase II experimental hut parameters (e.g. vector knock-down, mortality, blood feeding inhibition, and exiting) to predict malaria incidence/prevalence over time [2] and the impact of nets on malaria transmission. The WHO has ruled that such modelling is insufficient to judge new classes of LLINs at the present state of knowledge [10]. Malaria transmission dynamics simulated by such models have been used to predict the public health impact of different vector control interventions [17]. With further corroborative evidence, these models simulate transmission infection in populations of humans and mosquitoes, and could be used to extrapolate the results of cRCTs to other sites with different epidemiology, entomology, or mixtures of control interventions. It is unclear at present whether models parameterized with local hut trial data capturing the behaviour and survivorship of the local mosquito vector would be more accurate at predicting local epidemiology than this modelling approach which makes use of meta-analysis data from mosquito populations from disparate locations. A further drawback, these models have typically used data from LLINs subjected to 20 standardized washes to simulate the ageing process. As a result, their ability to predict is limited by the accuracy of standardized washing to reflect real life wear-and-tear and insecticidal durability under field conditions. In our studies, nets will be sampled from the community trials (at Phase III) for evaluation in experimental huts to parameterize the models.

The main aim of the study is to assess the insecticidal and physical durability of new dual-A.I. LLINs, Interceptor^®^G2, Olyset™ Plus and Royal Guard^®^ in the community over three years embedded within a cluster randomized controlled trial (cRCT). This is the first study assessing the durability of the novel dual-A.I. LLINs (Royal Guard^®^ and Interceptor^®^G2) and the synergist net, Olyset™ Plus. It is designed to support the development of bio-efficacy and physical durability criteria for partner A.I. in relation to the cRCT efficacy outcomes and refine preferred product characteristics developed by the WHO. Experimental hut trials done in the vicinity of the cRCT, with similar vector population characteristics using nets sampled from the main trial at intervals will allow us to understand the impact of field conditions, wear-and-tear and insecticidal deterioration on the efficacy of the dual-A.I. LLINs on entomological outcomes, and to relate these to the cRCT epidemiological and entomological outcomes.

A secondary aim is to establish whether entomological outcomes generated during the WHO product evaluation process (adapted experimental hut trials and supporting bio-efficacy testing) of nets sampled from the community can provide a proxy for epidemiological outcomes of cRCTs via transmission modelling.

## Methods

### Study area

**Fig. 1:**
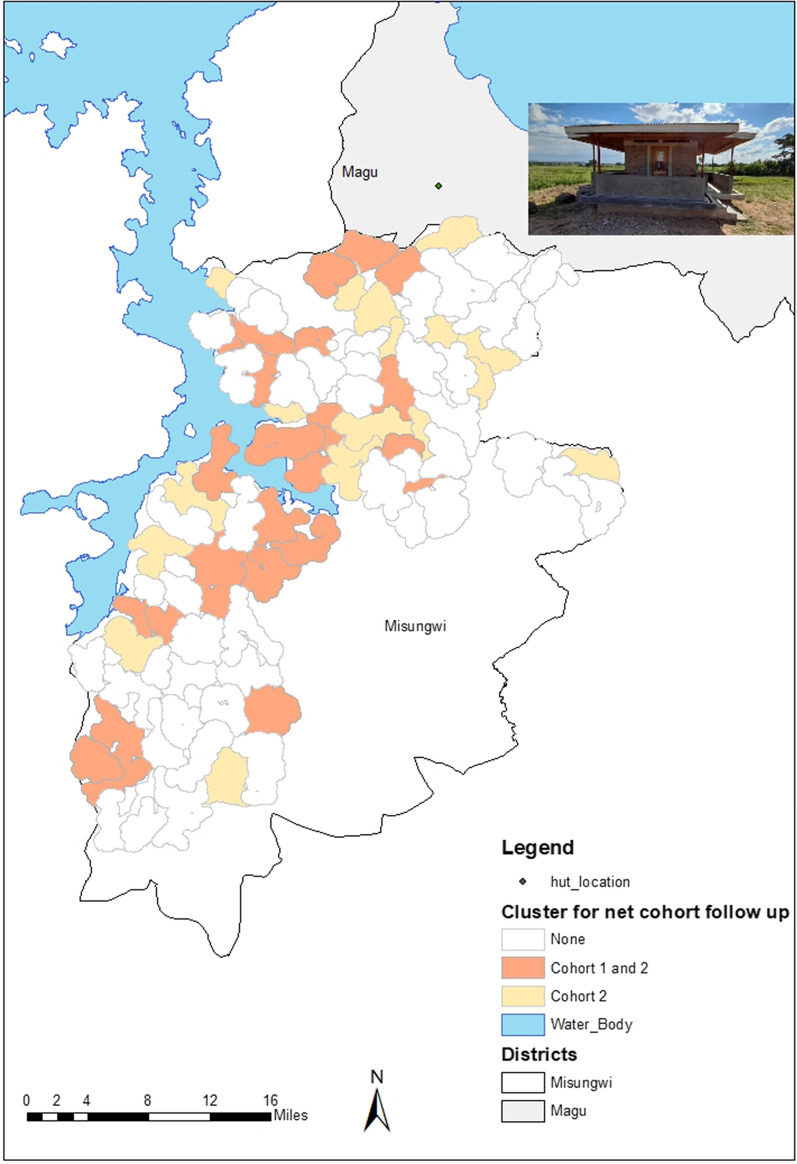
Map showing cRCT site and experimental hut site

The WHO Phase III durability study is part of a four-arm cRCT carried out in Misungwi district (2°51′00.0′′ S, 33°04′60.0′′ E), on the Southern border of Lake Victoria, Tanzania. The cRCT study area includes 72 villages, 42,314 households and a population of 251,155 based on a census done in 2018 as part of the study. A detailed description of the cRCT is provided elsewhere [11]. Six experimental huts are constructed in Magu district, Mwanza region, Tanzania (2°34.673′ S, 33°07.170′ E). The hut study site is north of the cRCT study area (Fig. 1). The main vectors in the study area are *Anopheles funestus *sensu stricto (*s.s*.), *Anopheles arabiensis* and *Anopheles gambiae s.s*., with *An. gambiae* s.s being the predominant species [18]*.*

### Descriptions of the interventions

The four LLINs under evaluation are (1) Royal Guard^®^, a net combining pyriproxyfen (PPF) known to disrupt female reproduction and fertility of eggs and the pyrethroid alpha-cypermethrin; (2) Interceptor^®^G2, a mixture net incorporating two adulticides with differing modes of action: chlorfenapyr and the pyrethroid alpha-cypermethrin; (3) Olyset™ Plus, a LLIN which incorporates a synergist piperonyl butoxide (PBO) to enhance the potency of pyrethroid insecticides; (4) Interceptor^®^ an alpha-cypermethrin only LLIN and the reference intervention (Table 1).

**Table 1:** Summary of the bioassay testing plan and outcomes per net type and mosquito strain

		Interceptor G2^®^	Royal Guard^®^	Olyset™ Plus
Susceptible strain: assessment of pyrethroid				
	Treatment	1/Untreated, 2/Interceptor, 3/Interceptor G2^®^	1/Untreated, 2/Interceptor, 3/Royal Guard^®^	1/Untreated, 2/Interceptor, 3/Olyset ™ Plus
Cone	Outcomes	Kd^a^, mortality 24, 48 and 72 h^b^	Kd, mortality 24, 48 and 72 h	Kd, mortality 24, 48 and 72 h
	Exposure time	3 min	3 min	3 min
	Total nets	30 nets (t0 to t30), 50 nets (t36)	30 nets (t0 to t30), 50 nets (t36)	30 nets (t0 to t30), 50 nets (t36)
	Total pieces	5 (t0) and 4 subsequent follow ups (position 2 to 5)	5 (t0) and 4 subsequent follow ups (position 2 to 5)	5 (t0) and 4 subsequent follow ups (position 2 to 5)
	Total replicates/piece	4	4	4
	Total Mosquito per test	5	5	5
	Total mosquitoes	Total between 4800 to 6000 per time point	Total between 4800 to 6000 per time point	Total between 4800 to 6000 per time point
Tunnel	Outcomes	Kd, mortality 24, 48 and 72 h, blood feeding	Kd, mortality 24, 48 and 72 h, blood feeding	Kd, mortality 24, 48 and 72 h, blood feeding
	Exposure time	12 to 15 h	12 to 15 h	12 to 15 h
	Total nets	All failed net with cone	All failed net with cone	All failed net with cone
	Total pieces	1 per net	1 per net	1 per net
	Total replicates/piece	2 replicates	2 replicates	2 replicates
	Total Mosquito per test	50	50	50
	Total mosquitoes	Determined by number of failing nets	Determined by number of failing nets	Determined by number of failing nets
Resistant strain: assessment of partner A.I. or synergist				
Cone	Outcomes		Kd, mortality 24, 48 and 72 h, ovarial development	Kd, mortality 24, 48 and 72 h
	Exposure time		3 min	3 min
	Total nets		30 nets (t0 to t30), 50 nets (t36)	30 nets (t0 to t30), 50 nets (t36)
	Total pieces		5 (t0) and 4 (t6 to t36)	5 (t0) and 4 (t6 to t36)
	Total replicates/piece		4	4
	Total Mosquito per test		5	5
	Total mosquitoes		Total between 4800 to 6000 per time point	Total between 4800 to 6000 per time point
Tunnel	Outcomes	Kd, mortality 24, 48 and 72 h, blood feeding	Kd, mortality 24, 48 and 72 h, Blood feeding, ovarial development	Kd, mortality 24, 48 and 72 h, blood feeding
	Exposure time	12 to 15 h	12 to 15 h	12 to 15 h
	Total nets	30 nets (t0 to t30), 50 nets (t36)	All failed net with cone	All failed net with cone
	Total pieces	1 per net (position 2)	1 per net	1 per net
	Total replicates/piece	2 replicates	2 replicates	2 replicates
	Total Mosquito per test	50	50	50
	Total mosquitoes	30 (50) nets × 1-piece × 2 replicates × 50 mosquitoes × 3 treatments = 9000	Determined by number of failing nets	Determined by number of failing nets
	Net Specificity	slow killing effect 72 h; mortality main outcome	Ovarial development by dissection at 72 h post exposure	Colony mosquito’s resistance strain (kdr^c^ and kdr + MFO^d^)

^a^Knockdown

^b^Hours

^c^Knockdown resistance

^d^Cytochrome P450 mono-oxygenase mechanisms

The four types of LLINs will be distributed to 84 clusters in the cRCT. Each household will receive one net for every two people. For odd numbers of occupants, the number of nets will be rounded up to cover the sleeping places. All nets distributed are rectangular (180 cm length × 160 cm width × 180 cm height) and dyed blue during manufacture. Forty of those clusters are selected for the durability study.

### Net durability assessment

**Fig. 2:**
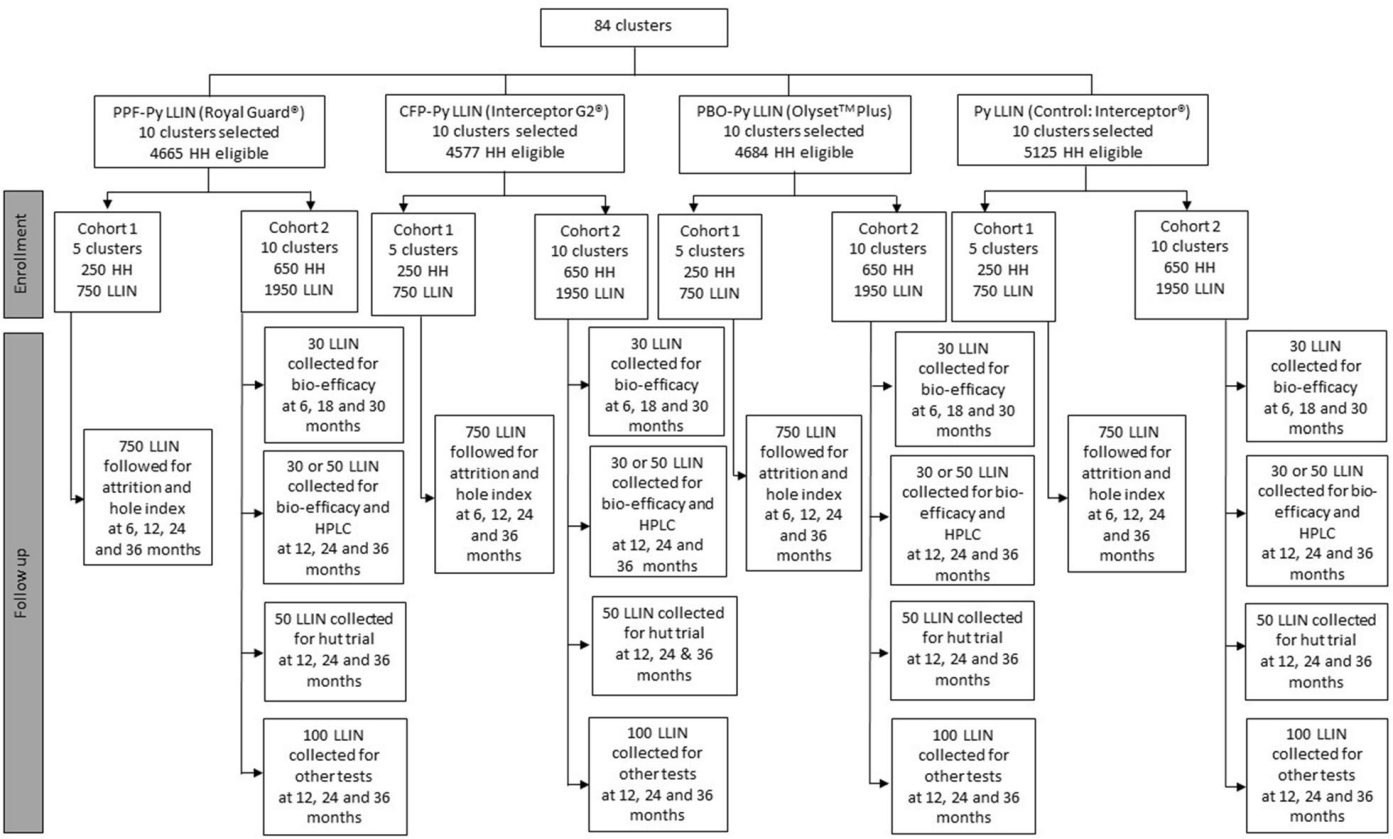
Study design flow chart

The efficacy and physical durability of the nets will be evaluated by means of a prospective cohort study (Fig. 2). A census/enumeration of the household in the hamlet is completed as part of the cRCT and for each house, name and GPS coordinates are available.

### Net attrition and fabric integrity (cohort 1)

A total of 250 households (HHs) in 5 clusters per study arm will be followed for LLIN physical integrity and attrition. Assuming an average of 3 LLINs per HH based on the average number of sleeping places, this will yield 750 LLINs. All 750 LLINs will be followed for attrition and for hole index. In each cluster HHs will be selected close to each other. During the first visit one month post distribution, in all the households willing to participate, their used LLIN will be identified with a unique identification number.

Household information on quality of housing and other socio-demographic characteristics will be collected (Additional file 1). The physical presence of all LLINs in the 250 HHs will be recorded at each visit; 6, 12, 24 and 36 months. If the net is still present in the HH, the investigator will record whether the net is being used for its intended purpose. Nets that are not used anymore will also be recorded. If the net is no longer in the house, the investigator will determine how it was lost. All LLINs followed for attrition and in current use will be inspected for number and size of holes on the side net panels divided into four areas from top to bottom and one on the roof position [19]. Size will be classified into four categories: smaller than a thumb (0.5–2 cm), larger than a thumb but smaller than a fist (2–10 cm), larger than a fist but smaller than a head (10–25 cm) and larger than a head (> 25 cm). Evidence of repairs to the net fabric and the type of repair will be recorded. Hole counts will be made by removing each net and arranging it over a frame and returning the nets after measuring physical integrity.

### Net withdrawal for insecticidal residual activity assessment and experimental hut trials (cohort 2)

LLINs will be withdrawn at 6 month time intervals up to 36 months and used for bio-efficacy testing and for once-per-year experimental hut trials. To reduce the impact of withdrawal on the cRCT outcomes, 10 clusters (5 clusters for attrition and integrity and 5 additional clusters for durability) per arm will be selected at random. A total of 650 HHs selected at random will give approximately 1950 LLINs per arm to follow up (Fig. 2). Each net collected will be replaced by a new net of the same type, but these new nets will not form part of the study. Households will remain part of the cohort until no cohort nets are available in the households. The unit of observation will be the individual net. As for cohort 1, selected nets for cohort 2 will be labelled and associated household information collected. From time t0 to t30, 30 nets per survey will be collected for bio-efficacy and 50 nets will be collected at t36 months. From each of the LLINs selected for bio-efficacy, one net piece measuring 30 cm × 30 cm will be cut from each side at baseline (t0 month), and in subsequent follow-ups only positions 2 to 5 will be cut, since position 1 situated at the bottom of the net may be disproportionately exposed to extreme abrasion when tucked under the bed, as per WHO guideline [9]. At each position, 3 samples adjacent to each other will be removed. The first will be used for chemical assay using High-Performance Liquid Chromatography (HPLC), the second for bio-efficacy on *An. gambiae s.s.* susceptible strain (Kisumu) and the third for bioassay on a resistant strain.

### Adverse events

For 200 HHs per intervention arm (20 HHs per cluster), perceived adverse effects from users or guardians of users will be recorded. The HH will be selected at random from all enrolled in cohort 1 and 2.

### Net bio-efficacy assessment

#### Mosquito strains

In the context of bioassay testing of dual-A.I. LLINs, WHO guidelines will be followed [9, 20]. As the former guideline pre-dates this protocol by several years and was focused mainly of pyrethroid LLIN, modifications are permissible to generate new evidence and were discussed with the WHO in advance. Bioassay testing of dual-A.I. LLINs will follow WHO guidelines [9, 20]. As the former published guideline pre-dates this protocol by several years and was focused mainly on pyrethroid LLIN, WHO-sanctioned modifications were permitted in advance to generate new evidence.

A susceptible *An. gambiae s.s.* strain (Kisumu) will assess the bio-efficacy of the pyrethroid in each of the dual-A.I. LLINs. To assess the durability (bioavailability) of the partner insecticide, it will be necessary to use a pyrethroid resistant strain or species ideally with resistance intensity great enough to withstand the effect of the pyrethroid. A*n. gambiae s.s.* Muleba-kis pyrethroid resistant strain (characterized by both *kdr*-East L1014S and mixed-function-oxidase based resistance) [21] will be used to assess the partner A.I. The resistant strain will be kept under constant pyrethroid selection pressure and phenotypic (using CDC bottle assay) and genotypic (molecular analysis by TaqMan assays) resistance will be monitored in every generation to assess changes in resistance frequency and intensity. The selection will be done once per generation at larval stage [21] using 0.08 µg/ml of alpha cypermethrin. Control larval bowls will be treated with 1 ml of ethanol.

#### Rationales for the four A.I.

In the dual-A.I. LLIN, Interceptor^®^G2, alpha-cypermethrin is fast-acting, and following a short exposure in contact bioassays, susceptible mosquitoes are knocked down within 1 h and dead within 24 h. The pyrrole chlorfenapyr requires longer exposure, is slower-acting taking up to 72 h to kill following contact bioassay; for monitoring delayed mortality, the WHO has proposed that mosquitoes may be held for 72 h with mortality reported every 24 h. There is clear evidence that cone bioassays with 3 min exposures fail to predict field efficacy of Interceptor^®^G2 [22]. WHO has stated that tunnel tests may be more appropriate to estimate the field durability of chlorfenapyr [23].

In the dual-A.I. LLIN Royal Guard^®^, PPF impacts egg development, while alpha-cypermethrin will induce mortality. The reproductive effects of PPF on blood-fed female mosquitoes are threefold. The first effect of PPF exposure is to disrupt the maturation of eggs and oviposition by females 2–3 days after blood-feeding. The second observable effect is reduction in the mean number of eggs per ovipositing female. The third effect is reduction in the hatch rate of laid eggs or in production of viable larvae. Conventionally, the effects on reproductive outcomes are assessed by observation of oviposition rate and hatch rate in mosquitoes exposed to PPF compared to unexposed mosquitoes [24, 25]. The problem encountered with oviposition as an indicator of fertility/sterility is the low oviposition rate in the pyrethroid-resistant control unexposed mosquitoes [26]. Furthermore, direct observations require a long follow-up, appropriate infrastructure, and can be laborious. An alternative approach is to dissect mosquitoes after exposure when eggs should normally have become fully mature (2–3 days post-blood meal). During the normal gonotrophic cycle, after taking a blood meal, the mosquito’s oocytes change in size and shape, and finally reach Christopher’s stage V which are a distinctive crescent shape [27]. Previous work on PPF-treated females has shown morphological defects on oocyte maturation in the ovaries following exposure, with development of PPF-affected oocytes arrested before reaching stage V [28].

In the dual-A.I. LLIN Olyset ™ Plus, PBO inhibits the cytochrome P450 oxidases responsible for metabolic pyrethroid resistance while permethrin will induce mortality. Cone tests have been used effectively to assess the performance of Olyset ™ Plus [29]; however, there is a need to use a resistant strain with oxidase-based mechanisms for these bioassays to assess the field durability of PBO.

#### Cone test

WHO cone tests will be performed on each of the pieces of the 30 (t0-t30) and 50 LLINs (t36) for the following products: Royal Guard^®^ and Olyset ™ Plus (both with susceptible and resistant mosquito strains) and Interceptor^®^G2 (only against susceptible strain) [9]. For each net sampled, all pieces cut will be tested. Cone will be set at an angle of 45° [30], four replicates will be done per net piece using 5 mosquitoes per cone and a total of 20 mosquitoes per piece, and 80 mosquitoes per net (100 for the baseline including position 1). For the control, an untreated net will be tested in parallel as well as 2 pieces of the standard LLIN collected at the same time point. Standard Interceptor^®^ LLIN will be used as a control. The estimated number of mosquitoes to be tested is detailed in Table 2.

**Table 2:** Study outcomes measurements

Outcomes	Measurements	Collection	Frequency
Bio-efficacy with susceptible and resistant *Anopheles* strains	1. Mortality recorded immediately (60 min) and after 24, 48 and 72 h post exposure 2. Blood feeding inhibition recorded in tunnel test 3. Fecundity/fertility for Royal Guard^®^ compared to standard LLIN and untreated net: proportion of abnormal ovaries 4. Blood feeding inhibition (%): the reduction in blood feeding of mosquitoes in the treatment compared with % feeding in the control tunnel	Cone or tunnel test on 4/5 pieces per net from 30 nets per type per time point and 50 nets at 36 months destructively sampled from cohort 2	At 0, 6^a^, 12, 18^a^, 24, 30^a^, 36 months post distribution
Net attrition rate (survival)	Household visit and observation of study LLIN presence: % of study nets that are lost (no longer in use for sleeping under) in the receiving household at each time point	Prospective cohort study cohort 1	At 6, 12, 24 and 36 months
Fabric integrity	Number of holes and hole size in study LLIN to calculate HI	Prospective cohort study cohort 1	At 6, 12, 24 and 36 months
Chemical content	HPLC of pyrethroid and partner A.I.: amount of active ingredients in fiber	bio-efficacy	At 0, 12, 24 and 36 months
Adverse effect	Household visit and questionnaire to report: skin itching, facial burning, sneezing, nose running, headache, nausea, eye irritation, other	Prospective cohort study cohort 1 and 2 nets	At one month post distribution
Efficacy against free flying mosquitoes	1. Immediate mortality (%) recorded and after 24, 48 and 72 h 2. Blood feeding inhibition (%): the reduction in blood feeding of mosquitoes in the treatment compared with % feeding in the control huts 3. Deterrency (%): reduction in hut entry relative to the control huts with untreated nets 4. Exophily (%): The proportion of mosquitoes that exit early and are found in exit traps compared with the untreated control 5. Personal protection (%): the reduction in the number of mosquitoes blood-fed compared to number of mosquitoes blood-fed in the untreated control 6. Fecundity reduction (%): the reduction in fecundity per blood-fed female alive at 72 h after exposure (using dissection methods) in Royal Guard^®^ compared to standard LLIN and untreated net	1. Adapted experimental hut at each time point 2. Standard wash resistance experimental hut (0 and 20 wash nets)	1. At, 0, 12, 24 and 36 months 2. Once during the course of the trial

*h* hours, *HI* hole index, *HPLC* high-performance liquid chromatography

^a^Test only performed when dual A.I. does not meet WHO criteria of > 80% mortality, 24 h post exposure and does not show superior efficacy compared to standard LLIN against resistant *Anopheles* at the yearly time point

Per bioassay, five unfed 2–5 days old *An. gambiae s.s.* will be introduced into each cone. After 3 min exposure, mosquitoes will be transferred into labelled paper cups covered with untreated netting with access to 10% sugar solution. The bioassays will be carried out at 25 ± 2 °C and 75 ± 10% RH and at 27 ± 2 °C. Any net which fails the cone criteria, i.e. mortality < 80% and sterility < 60% with pyriproxyfen exposure will be re-tested using the tunnel test.

#### Tunnel test

To assess the residual bio-efficacy of the chlorfenapyr component of Interceptor^®^G2, the tunnel test using resistant mosquitoes shall be specifically used as the first choice bioassay in preference to the cone test owing to its unusual mode of action on flight muscle function [22]. The piece of net in position 2 of each of the 30 Interceptor^®^G2 will be systematically tested in the tunnel (Table 1). To assess the pyrethroid component of Interceptor^®^G2, the cone test is suitable using the pyrethroid susceptible strain as the first-choice bioassay.

The tunnel test will be also used to assess the residual bio-efficacy of any LLIN pieces (Royal Guard^®^ and Olyset ™ Plus) that do not meet the criteria of ≥ 95% knockdown (KD) after 60 min or mortality of ≥ 80% after 24 h in cone bioassays. For failing nets of Royal Guard^®^ and Olyset ™ Plus, the net piece that produces mortality closest to the mean mortality during the cone test will be used in the tunnel test.

The procedure for use of guinea pigs will be compliant with criteria laid down in EC Directive 86/609/ECC concerning protection of animals used for experimental purposes. The animal ethics approval has been sought from LSHTM. The glass tunnel is 25 cm^2^ and 60 cm long, divided at one third of the length by a disposable cardboard frame to which the LLIN netting piece is attached. The surface of netting “available” to mosquitoes is 400 cm^2^ (20 cm × 20 cm). Nine holes, each 1 cm in diameter, (one at the center of the square and the other eight equidistant at 5 cm from the border) will be made in the netting to allow for passage of mosquitoes. Netting-covered cages at both ends provide easy access to add and remove mosquitoes. In one cage, a guinea pig will be restrained. Fifty unfed 5–8 days old mosquitoes will be introduced at the opposite end of the tunnel from where the guinea pig is restrained. The experiment will begin at 18:00 and end at 08:00 the following morning, mosquitoes will be scored according to whether they passed through the netting, whether they successfully blood fed and whether they survived the exposure period.

#### Cylinder test

The cone and tunnel tests may prove inadequate for evaluating the durability of partner A.I.s over 1–3 years once the insecticidal content starts to decrease. The standard cone test exposes mosquitoes for 3 min only, which has been shown to underestimate contact time and the exposure time and mortality attained in experimental huts using resistant mosquitoes; 30 min exposure is probably more realistic when using resistant mosquitoes [31]. Mortality generated with free-flying resistant mosquitoes in experimental huts correlates well with mortality attained in 30 min bioassay for some insecticides tested, e.g., chlorfenapyr and Interceptor^®^G2 (Rowland and Kirby unpublished data). While the contact time may differ for other insecticides or when the concentration decreases during 3 years in the field; now that the precedent is established for one type of dual-A.I. LLIN, the average contact time of free flying mosquitoes is likely to be longer than 3 min for other nets too. The WHO cylinder test will be performed for Olyset™ Plus, Royal Guard^®^ and Interceptor^®^G2 on a sub-sample of nets at each time point and compared to tunnel and cone results.

The netting will be stapled to WHO control test papers measuring 15 cm × 12 cm to facilitate rolling and fitting into the test cylinder in the same way as an insecticide test paper would be fitted. Holding rings are inserted to hold back the netting [32]. Bioassays will follow the same procedure as insecticide susceptibility testing except that exposure time will be 3, 15, 30 and 60 min as necessary and this will be recorded as knockdown. Before exposure, 10 mosquitoes will be aspirated into the holding cylinder of the kit and then blown into the exposure cylinder according to standard procedures. After exposure, the test insects are blown back into the holding cylinder and 10% sugar solution provided. Ten mosquitoes per cylinder test would ensure a density per unit area of netting similar to that of five mosquitoes per cone.

### Experimental hut design

The experimental huts in Magu are a modified version of the standard East African hut [33] featuring four brick walls, a wooden ceiling lined with hessian sackcloth, an iron sheeting roof, two baffled eave gaps above each wall, and a window trap on each wall. The huts are built on concrete plinths and surrounded by a water-filled moat to deter entry of scavenging ants. In the modified design, the four verandas are open; the baffled eave gaps above all four sides allow unimpeded entry of mosquitoes and minimal mosquito exiting. Mosquitoes are restricted to exiting through the window traps on the four walls of the hut. In each hut, cloth sheets are laid on the floor each night to ease the collection of knocked-down mosquitoes in the morning. Sugar solution is provided at night in the window traps to reduce mosquito mortality.

The nets will be evaluated using experimental huts for their effects on free-flying, wild *An. gambiae *sensu lato (*s.l*.), *An. funestus s.l.* and *Culex quinquefasciatus* mosquitoes for their ability to deter entry, repel mosquitoes, induce mortality and inhibit blood-feeding.

### Adapted experimental hut study

Each of the 30 individual nets per product type collected from community at t12, 24 and 36 months will be tested in experimental huts. The following treatments shall be assessed at each time point:

1. Control: untreated polyethylene net with 6 holes.
2. Standard LLIN: new Interceptor^®^ washed one time with 6 holes.
3. Interceptor^®^ at t12/t24/t36.
4. Interceptor^®^G2 at t12/t24/t36.
5. Royal Guard^®^ at t12/t24/t36.
6. Olyset™ Plus at t12/t24/t36.

The study will be done over 6-week periods. Sleepers will be rotated between huts on successive nights to account for individual attractiveness and net treatments rotated every week following a random Latin square design (Additional file 3). Every week, collections shall be performed over 6 days and on the last day huts will be cleaned and aired before the next treatment rotation. Six replicates of untreated net and of new standard LLIN will be tested per hut treatment and will be swapped every day within each week of the trial. Field collected nets will be changed every day and tested for one night only per trial. Because there are 36 day/night collection per treatment for a complete Latin square rotation (sleepers and treatments) and only 30 field collected individual nets per product type an additional 6 new LLIN from each treatment will be evaluated (treatment 3 to 6). These new nets will act as a positive control and variation in outcomes over time in those nets will be accounted for in the analysis comparing efficacy of field net between time points. Six holes of 4 × 4 cm will be cut in the untreated and new LLINs used in each treatment arm following WHO guidelines [9]. Hole size will be counted for the field collected nets as per cohort 1 nets, and followed for fabric integrity. A hut trial study for each time point will be repeated 2 to 4 times to account for vector composition seasonality.

### Mosquito processing

All mosquitoes collected in experimental huts will be monitored for three days and mortality recorded after 24, 48 and 72 h post collection. Blood fed mosquitoes collected from Royal Guard^®^, Interceptor^®^ and untreated nets, will be dissected after 72 h for observation of fertility/sterility. Mosquitoes will be recorded as fertile if the eggs are fully developed into Christopher’s stage V (Additional file 2).

After dissection the first reader will read the slide and enter the results in a form, then a second reader will record the results in separate forms. All information will be entered into a database in Access and compared for consistency. If there is variation between readings, the third reader will be assigned to read the slide and record the results. All slides will be kept in fridge until the results has been confirmed by data manager.

A subset of live and dead mosquitoes from each hut will immediately be killed (if still alive) and stored in RNAlater^®^ at − 80 °C for species identification and resistance gene expression analysis. Following molecular species identification, presence/absence of resistance alleles will be compared between individuals of assumed resistant (alive) and susceptible (dead) phenotypes and changes in allele frequency will be compared between hut conditions and between baseline characterization and post-intervention.

### Modelling of experimental hut trial entomological surrogates

While experimental hut trials are the gold standard for assessing LLIN efficacy against susceptible and resistant mosquitoes, mathematical modelling [2] can be used to predict the public health impact of factors such as pyrethroid resistance on LLIN efficacy and malaria transmission [2]. The models are calibrated to the local area using site-specific entomological and epidemiological data collected from the cRCT site (such as baseline mosquito bionomics, history of LLIN use and baseline malaria prevalence). Two sets of parameters are used to characterize the efficacy of trial dual-A.I. LLINs. The first uses estimates of the proportions of mosquitoes dying, blood-feeding and outcomes such as deterrence, exiting and repellence estimated using experimental hut trials conducted in the region of the cRCT. The second uses estimates for the same metrics derived from a meta-analysis of all currently available experimental hut trial data for the same dual-A.I. LLINs from across Africa. Models parameterized with these two sources of data are used to predict changes in malaria prevalence over time. These two models are statistically compared to the observed results of the cRCT at different time points following the mass campaign to investigate the benefit of local LLIN efficacy information. WHO discriminating dose bioassays are used to quantify the frequency of resistance in the mosquito populations in the vicinity of the experimental hut site to provide a link between the outcomes of the trial and widely used assays for assessing the frequency of resistance.

There is considerable uncertainty in how the efficacy of nets changes over time. It can be estimated using the WHO proxy of standardized washing, as described by experimental hut outcomes from trials evaluating LLINs washed 0 and 20 times. The number of washes taken to halve the killing activity of the LLIN can be estimated and converted into predictions of the insecticidal half-life in years considering 20 washes to represent the decay expected in an LLIN over three years of use in the field. Estimates of the actual duration of insecticidal activity in the field is one of the more uncertain features of experimental hut trials and consequently for models evaluating the public health impact of novel LLINs. This is because of the uncertainty in the relationship between the number of washes and durability of insecticide in the field over time for non-pyrethroid A.I.s. Under field conditions, nets are subject to many environmental factors affecting durability in addition to washing, such as friction, wood smoke, and everyday wear and tear. This uncertainty is important to include as the average age of LLINs in Africa is over one year old and small changes in LLIN-induced mortality over time can have a large epidemiological impact as population LLIN coverage falls two or three years after the last mass distribution campaign. This problem seems particularly acute for Interceptor^®^G2 as chlorfenapyr cannot be evaluated in simple cone bioassays and nets washed 20 times [34]. Evaluation of naturally aged LLINs collected from the field in experimental hut trials may allow more precise predictions of the longevity of a LLIN (i.e. half-life), allowing the decay in insecticidal activity to be directly estimated instead of having to rely on proxy measures. This is anticipated to improve the accuracy of epidemiological predictions made from entomological data which can be evaluated by comparing model predictions to the results of the main cRCT. The utility of incorporating other entomological data collected as part of the trial into the modelling framework shall be investigated.

## Results

The study outcomes are summarized in Table 2.

### Sample size calculation

Sample size calculations for prospective LLIN study of net survivorship were performed using the power log rank command in Stata v.15.1. A total of 750 LLINs per type from 5 clusters per arm (i.e. 150 per cluster) will allow detection of a 9.4% absolute difference (hazard ratio = 0.8651) in LLIN attrition rate assuming an attrition rate in the control arm of 70% over the 3 years. This is assuming an intra-cluster correlation coefficient (ICC) of 0.03. A hazard ratio = 0.7951 (i.e. 14.3% absolute difference) can be detected, assuming an ICC = 0.01, and a hazard ratio = 0.7478 (i.e. 17.7% absolute difference) for an ICC = 0.02.

### Data management

All data on LLIN physical conditions, washing and household characteristics will be collected using Open Data Kit (ODK) forms. Bioassay data will be recorded on standardized forms and double entered into an Access file and linked to the database via the net identification number and time interval. Consistency checking will be done by running algorithms especially designed to identify sources of error. This database will be sent by the data manager to the project manager after each time-interval. The project manager will keep an updated master list of the location and status of each net. Data from bioassay and chemical residue analysis will be entered separately. Hut data will be entered directly in electronic forms prepared in ODK. Standard Operating Procedures (SOPs) for data collection will be developed and field staff will be appropriately trained to ensure rigorous data collection. This will include quality control (QC) of their own performance by checking for missing data or implausible responses. Further QC will be conducted by a supervisor who monitors performance of field staff by checking for completeness and internal consistency of responses within hours of data collection. To maintain participant confidentiality, all consents forms will be kept in a locked cabinet only accessible by an authorized staff. Statistical analysis will be performed using Stata software v.15.1.

### Data analysis

#### Fabric integrity (hole index)

Physical integrity of each net will be measured by the Hole Index (HI) as per WHO guideline [9]. Holes will be counted and only the hole size > 0.5 -2 cm will be recorded as size 1, 2–10 cm will be size 2, 10–25 cm size 3 and > 25 cm in diameter will be size 4 [9]. HI will be calculated using the formula HI = (1 × no. of size-1 holes) + (23 × no. of size-2 holes) + (196 × no. of size-3 holes) + (576 × no. size-4 holes). Based on the HI the nets will be divided into 3 categories: (1) Good: HI < 64 (hole surface area < 79 cm^2^. (2) Acceptable: HI = 64  − 642 (80–789 cm^2^), (3) too torn; HI > 642 (hole surface area > 790 cm ^2^ [35]. Mean and median hole index and surface will be reported by type of net as well as proportion nets in each hole categories (good-acceptable and too torn). Negative binomial regression models will be used to compare hole surface area between net types including co-variates as socio economic status, housing and sleeping conditions.

#### Attrition/survival

The rate of attrition will be first calculated as the proportion of study nets lost among all study nets originally received and further divided into reasons of net loss. To estimate functional survival only the attrition due to destruction, discarding or use for other purpose will be included (MPAC recommendation) [37]. The functional survival rate (pX) of each type of study net at each time point will be calculated as:

$$\begin{array}{l} {\mathbf{pX}} = \% \;{\text{surviving}\; \text{to}\; \text{time}\; \text{X}} \\ \qquad = \left( {{\text{number}\; \text{of}\; \text{study}\;\text{net}\; \text{present}\; \text{and}}\;{``}{\text{serviceable}}{"}\;{\text{at}\;\text{time}\;\text{X}}/{\text{number}\;\text{of}\;\text{study}\;\text{net}\;\text{originally}\;\text{received}\;\text{and}\;\text{not}\;\text{given}\;\text{away}\;\text{at}\;\text{time}\;\text{X}}} \right) \, \times 100. \end{array}$$

Descriptive statistics will be used to present the proportion surviving for each study net at each time point and compare the dual-A.I. LLINs to the standard LLIN. Cluster effect will be accounted for to estimate the 95% confidence interval.

#### Median survival time

Median survival time is the time point at which 50% of the study nets received are still present and in serviceable condition. First the functional survival rate to time X will be compared against a reference survival curve provided by WHO in the VCTEG report [35] The functional survival rate to time X will also be compared between the dual-A.I. LLINs against the standard LLIN. A Kaplan–Meier survival analysis will be used to estimate the median survival time of each study net and will consider the design effect.

#### Efficacy and long-lasting effect of the pyrethroid

The dual-A.I. net will meet WHO criteria [9] for efficacy and long-lasting effect of the pyrethroid if, after 36 months of use, at least 80% of sampled nets tested against a pyrethroid susceptible mosquito strain are effective in WHO cone tests (≥ 95% knockdown or ≥ 80% mortality after 24 h) or tunnel tests (≥ 80% mortality or ≥ 90% inhibition of blood-feeding). Thresholds for the second A.I. of each dual-A.I. LLIN are not known yet and would need to be established in relation to the cRCT findings. However, dual-A.I. LLIN efficacy outcomes against the resistant *Anopheles* strain will be compared to those of standard LLIN to assess superior effect at each time point.

#### Experimental hut trial analysis

Proportional outcomes (blood-feeding, exiting and mortality, oviposition) related to each experimental hut treatment will be assessed using binomial generalized linear mixed models (GLMMs) with a logit link function. A separate model will be fitted for each outcome. In addition to the fixed effect of each treatment and hole index categories, each model will include random effects to account for the following sources of variation: between the huts; between the sleepers; between the weeks of the trial; between trial rounds; and finally, an observation-level random effect to account for variation not explained by the other terms in the model (over dispersion). Location of the holes (zone 1–5) in relation of mosquito blood feeding and net entry will also be explored.

Differences in deterrence, proportions of adult females killed, proportions blood-fed, overall killing effect, personal protection and number of females laying eggs between the treatments will be analysed using negative binomial regression based on numbers entering, numbers blood-fed, killed and numbers observed for oviposition, respectively, with adjustment for the above-mentioned covariates.

Entomological data from the entomological surrogate studies (experimental hut trials and supporting efficacy bioassays) will be integrated into a meta-analysis and modelling of disease outcomes to investigate possible relationships between epidemiological and entomological data from the cRCT. Hole count data will be weighted as per WHO guidelines and the results compared with the recorded mortality per net type.

Comparisons of the changes in levels of gene expression will be performed between live mosquitoes collected from experimental huts containing treated interventions and experimental huts with no treatment/control treatment (relative to a susceptible laboratory control colony). The assumptions are that comparing between these two experimental groups will allow us to differentiate between metabolic genes which are constitutively over-expressed in the field populations and may contribute to resistance (i.e. expression levels observed in the control hut which contains a mixture of ‘resistant’ and ‘susceptible’ vectors of unknown phenotype) and those genes which are over-expressed in our field populations in response to intervention/insecticide exposure (i.e. expression levels observed in the treated huts compared to the control huts).

## Discussion

The WHO Phase I, II and III is a gated process which makes assumptions about the relationship between durability over time and artificial washing as done in the laboratory. The 20 washes of Phase I and II testing is intended as surrogate for insecticide durability which may or may not correlate with laboratory and experimental hut assays on nets taken from the field over the 3 years of a WHOPES/WHO-PQ Phase III longitudinal study, nor with modelled outputs of nets taken from the field. For example, under field conditions the nets are subject to levels of abrasion which standardized washing using a bucket and pole cannot match [8]. Nets used in standardized Phase II studies are deliberately cut to make 6 holes; the average number of holes in field collected nets may exceed 20 after less than 2 years.

In large-scale field trials, physical durability is affected by net care and repair, frequency of use and maintenance practices, duration of transmission season, as well as textile physical features such as fibre material, knitting or weaving pattern [36]. The WHO assumes a good LLIN will demonstrate a physical life span of 3 years but this duration will vary between product, endemic regions and condition of use [37, 38]. In the WHO guidelines, LLIN survivorship and fabric integrity are monitored in the community at 6, 12, 24 and 36 months [9, 37]. The pyrethroid component is expected to remain effective for 3 years [9] while the residual efficacy of other active ingredients are not yet known [37, 38]. The proposed work was designed to address those assumptions and uncertainties, to establish the true correlations between WHOPES Phase I, II and III and improve the fit of transmission models to cRCT outcomes [37].

The data will help develop bio-efficacy and physical durability criteria for partner A.I.s, in relation to the cRCT epidemiological and entomological outcomes, and refine preferred product characteristics of each class of LLIN. Data generated by the study will be used to parameterize transmission models which in turn will be used to predict epidemiological outcomes after various intervals of use. Comparison of predicted outcomes with actual cCRT outcomes will determine the accuracy of the models and whether experimental hut testing of nets sampled during longitudinal modelling could serve as a surrogate for cCRTs. Since cCRT cannot be replicated in every location, the outcome of the study may show minimum standards the study net should meet in other places using experimental hut and modelled data.

Data generated by this study may elaborate the criteria or new thresholds of performance for new edition of the LLIN guidelines to help evaluate second in-class Dual A,I, LLIN products in these categories [10].

## Supplementary Information

**Additional file 1.** Net follow up questionnaire.

**Additional file 2.** Standard Operation Procedure (SOP) for dissection and estimation of oviposition inhibition.

**Additional file 3.** Random Latin Square DO file (STATA 15.1).

**Additional file 4.** Net Study informed consent agreement.

**Additional file 5.** Volunteers information sheet and consent agreement (Hut trial).

## Data Availability

Not applicable.

## Abbreviations

A.I.: Active Ingredient
CDC: Centers for Disease Control and Prevention
cRCT: Cluster Randomized Control Trial
GPS: Global Positioning System
HH: Household
HI: Hole Index
HPLC: High-Performance Liquid Chromatography
KCMUCO: Kilimanjaro Christian Medical University College
LLIN: Long Lasting Insecticidal Net
LSHTM: London School of Hygiene and Tropical Medicine
MRCC: Medical Research Coordinating Committee
NIMR: National Institute for Medical Research
ODK: Open Data Kit
PBO: Piperonyl butoxide
PPF: Pyriproxyfen
QC: Quality Control
SOP: Standard Operating Procedure
WHO: World Health Organization
WHOPES: WHO Pesticide Evaluation Scheme
WHO-PQ: WHO Prequalification Programme
